# Editorial: Systemic involvement in obstructive sleep apnea: Personalized medicine to improve health outcomes

**DOI:** 10.3389/fmed.2022.999977

**Published:** 2022-08-16

**Authors:** Gonzalo Labarca, Manuel Sanchez-de-la Torre, Jorge Jorquera

**Affiliations:** ^1^Division of Sleep and Circadian Disorders, Brigham and Women's Hospital and Harvard Medical School, Boston, MA, United States; ^2^Precision Medicine in Chronic Diseases, Hospital Universitari Arnau de Vilanova-Santa Maria, IRB Lleida, Faculty of Nursing and Physiotherapy, University of Lleida, Lleida, Spain; ^3^Department of Medicine, Centro de Investigación Biomédica en Red de Enfermedades Respiratorias (CIBERES), Madrid, Spain; ^4^Grupo de Estudio Trastornos Respiratorios del Sueño (GETRS), Centro de Enfermedades Respiratorias, Clinica Las Condes, Santiago, Chile

**Keywords:** obstructive sleep apnea, sleep apnea, sleep apnea and cardiovascular diseases, precision medicine, hypoxemia

Obstructive sleep apnea (OSA) is a frequent condition with a high impact on health care. The estimated prevalence of OSA range between 20 and 40%, and more than one billion patients are at risk of OSA worldwide (1). Current OSA diagnosis is achieved by the Apnea-Hypopnea index (AHI), and moderate to severe OSA is strongly associated with worse outcomes (2). Although snoring, witnessed apneas, and excessive sleepiness during the day are the main OSA-related symptoms, OSA is frequently non-diagnosed in clinics and, therefore, undertreated (3). Additionally, OSA is widely associated with various systemic comorbidities related to an increased risk of poor incident outcomes at follow-up, especially in non-sleepy patients. The main OSA—related comorbidities are cardiovascular disease, cardiometabolic, and increased risk of prevalent and incident cognitive impairment and cancer diagnosis (4, 5).

In this scenario, a precision-medicine approach including the different OSA phenotypes, incorporating the burden of comorbidities, is a significant issue for researchers and health care providers to improve health outcomes (6–8). According to clinical guidelines, the gold standard treatment for moderate to severe OSA is continuous positive airway pressure (CPAP) (9). However, randomized controlled trials aimed to explore the efficacy of CPAP on OSA comorbidities and cardiovascular outcomes have failed to show significant efficacy in improving secondary cardiovascular prevention (10–13). A potential explanation for these non-consistent results is the inadequate selection of patients for these trials using AHI (14). Therefore, identifying novel measures, including genetics, biomarkers, clinical features, and signals derived from sleep study tests to better determine a high-risk OSA phenotype of worse health outcomes are relevant.

## A novel approach to identifying OSA and their prognosis

In this Research Topic, we included novel data on the association between OSA and cardiovascular and metabolic outcomes. We also had data from a novel approach to better identify a population with OSA and a high risk of cardiovascular complications. Zapater et al. on: “*Respiratory polygraphy patterns and risk of recurrent cardiovascular events in patients with acute coronary syndrome,”* following a machine-learning approach, developed and validated a high-risk pattern of a recurrent cardiovascular event following a principal component analysis including conventional HSAT metric [AHI, oxygen desaturation index, mean and minimum oxygen saturation (SaO_2_), the average duration of events and percentage of time with SaO_2_ < 90% (T90%)] in two different datasets including data from the ISAACC randomized controlled trial (10) (training dataset) and the community-based cohort study (HypnoLaus) (15). As a result, a high-risk profile of patients can be identified, with an increased risk of recurrent cardiovascular events.

Moreover, genetics is another crucial component of the precision medicine approach, and intermittent hypoxia can trigger changes across different physiopathology pathways. In their study, Wu et al., “*The discovery, validation, and function of hypoxia-related gene biomarkers for obstructive sleep apnea,”* determined the diagnostic value of hypoxia-related genes, exploring their potential molecular mechanisms of action in OSA. As a result, 16 genes associated with hypoxia showed a high predictive value, and four of them may be related to OSA *via* inflammatory pathways and, interestingly, may contribute to OSA-related cancer risk.

## Impact of intermittent hypoxia in the liver

The liver is a critical component in regulating and expressing several pro-inflammatory pathways, and intermittent hypoxemia can influence their expression and, therefore, the clinical impact in different tissues (5, 16). In their study, Gaucher et al., “*Intermittent hypoxia rewires the liver transcriptome and fires up fatty acids usage for mitochondrial respiration,”* using an animal model exposed to nocturnal hypoxemia, determined that nocturnal hypoxemia increases the oxidative capacity from fatty acids of liver mitochondria, increased the hepatic production of oxidative stress markers. Clinically, liver damage can be associated with non-alcoholic fatty liver disease, Landete et al.: “*Increased oxygen desaturation time during sleep is a risk factor for NASH in patients with obstructive sleep apnea: A prospective cohort study”* in a prospective study including 153 subjects with OSA and 43 non-OSA population, reported a high prevalence of hepatosteatosis and NASH in OSA patients than controls. Moreover, male gender, high body mass index, diabetes, and higher T90% (defined as >10%) were associated with NASH.

## Impact of OSA on long-term outcomes

Cardiovascular disease is associated frequently with OSA, and managing OSA and heart failure is critical to improving long-term outcomes. Herein, Wang et al., on: “*Management of obstructive sleep apnea in patients with heart failure”* published an updated state-of-the-art review including data from previous and novel studies aimed to test different therapies for OSA with Heart failure, including both surgical and non-surgical alternatives and future research in this field.

Gender difference and OSA among women are frequently underrepresented (17, 18). In this topic, Alonso-Fernandez et al., on: “*Influence of obstructive sleep apnea on systemic inflammation in pregnancy*,” evaluated a total of 11 pregnant women in the third trimester with OSA (AHI ≥ five events/h) compared with 22 controls (pregnant women at third trimester without OSA), the main finding was the significant association between inflammatory cytokines TNF-α, IL-1β, IL-8 with OSA and higher T90% (a marker of nocturnal hypoxemia) (19). Additionally, systemic inflammation was inversely correlated with neonatal birth weight and age.

Finally, the association between sleep health and OSA after coronavirus-19 (COVID-19) infection is a novel and exciting field to explore in future research (20, 21). In an original article published by Labarca et al.: “*Impact of Obstructive Sleep Apnea (OSA) in COVID-19 Survivors, symptoms Change between four months and one year after the COVID-19 infection.”* including a nested cohort of 60 patients survivors from COVID-19, with a follow up of 12 months, the association of untreated OSA was associated with an increased expression of pro-inflammatory cytokines IL-6, and clinically, worse neurocognitive and metabolic outcomes across different COVID-19 severities during the acute period.

In sum, there is extensive evidence that OSA is widely associated with multisystemic outcomes and novel data, including OSA-driven metrics, especially data from markers of nocturnal hypoxemia, in addition to biomarkers and following a precision medicine approach will be a keystone in the incoming years. Prompt identification of the OSA population and their severity should decrease the risk of worse health outcomes.

## Author contributions

GL contributed to data analysis and drafting of the manuscript. MS-d-l and JJ contributed to the study and critical revision of the manuscript. All authors contributed to the interpretation of the data, critical revision of the manuscript, and approved this manuscript in its final form.

## Funding

GL declares grant support from the National Institute of Health (1R21HL161766-01) and the American Academy of Sleep Medicine (254-FP-21).

## Conflict of interest

The authors declare that the research was conducted in the absence of any commercial or financial relationships that could be construed as a potential conflict of interest.

## Publisher's note

All claims expressed in this article are solely those of the authors and do not necessarily represent those of their affiliated organizations, or those of the publisher, the editors and the reviewers. Any product that may be evaluated in this article, or claim that may be made by its manufacturer, is not guaranteed or endorsed by the publisher.
